# Molecular Characterization and Comparative Sequence Analysis of Defense-Related Gene, *Oryza rufipogon* Receptor-Like Protein Kinase 1

**DOI:** 10.3390/ijms13079343

**Published:** 2012-07-24

**Authors:** Yee-Song Law, Ranganath Gudimella, Beng-Kah Song, Wickneswari Ratnam, Jennifer Ann Harikrishna

**Affiliations:** 1 Centre for Research in Biotechnology for Agriculture (CEBAR) and Institute of Biological Sciences, Faculty of Science, University of Malaya, Kuala Lumpur, 50603, Malaysia; E-Mails: yeesong0210@yahoo.com (Y.-S.L.); ranga@siswa.um.edu.my (R.G.); 2 School of Science, Monash University Sunway Campus, Jalan Lagoon Selatan, Bandar Sunway, Selangor 46150, Malaysia; E-Mail: song.beng.kah@monash.edu; 3 School of Environmental and Natural Resource Sciences, Faculty of Science and Technology, Universiti Kebangsaan Malaysia, Bangi, Selangor 43600, Malaysia; E-Mail: wicki@ukm.my

**Keywords:** *Oryza rufipogon*, RPK1, LRR domain, kinase domain, disease resistance

## Abstract

Many of the plant leucine rich repeat receptor-like kinases (LRR-RLKs) have been found to regulate signaling during plant defense processes. In this study, we selected and sequenced an *LRR-RLK* gene, designated as *Oryza rufipogon receptor-like protein kinase 1* (*OrufRPK1*), located within yield QTL *yld1.1* from the wild rice *Oryza rufipogon* (accession IRGC105491). A 2055 bp coding region and two exons were identified. Southern blotting determined *OrufRPK1* to be a single copy gene. Sequence comparison with cultivated rice orthologs (*OsI219RPK1, OsI9311RPK1* and *OsJNipponRPK1,* respectively derived from *O. sativa* ssp. *indica* cv. MR219, *O. sativa* ssp. *indica* cv. 9311 and *O. sativa* ssp. *japonica* cv. Nipponbare) revealed the presence of 12 single nucleotide polymorphisms (SNPs) with five non-synonymous substitutions, and 23 insertion/deletion sites. The biological role of the *OrufRPK1* as a defense related LRR-RLK is proposed on the basis of cDNA sequence characterization, domain subfamily classification, structural prediction of extra cellular domains, cluster analysis and comparative gene expression.

## 1. Introduction

Plant receptor-like kinases (RLKs) form one of the largest gene families and are grouped within the RLK/*Pelle* family. To date, a large number of RLK*/Pelle* family members have been reported for *Arabidopsis thaliana* (1027) and rice (1429) [1]. In the plant kingdom, the RLK/*Pelle* family can be sub-classified into more than 40 subfamilies based on their sequences and structural analysis [2,3]. Amongst the groups, leucine-rich repeat (LRR)-like transmembrane receptor kinases represent the largest subfamily of the plant RLK*/Pelle* family. These proteins contain an extracellular domain connected to a cytoplasmic serine/theronine protein kinase domain via a single pass transmembrane helix [3]. In general, plant LRR-RLK family members play a vital role in plant defense or developmental related pathways by perceiving extracellular signals such as plant hormones or pathogen-associated molecular patterns (PAMPs) respectively.

Nevertheless, only a handful of LRR-RLKs are well studied in plants. These reported LRR-RLKs include candidates which have been associated with plant development in numerous species such as Floral Organ Number 1, FON1 [4]; Commissural Vein Excessive 1, COE1 [5] and Leucine-rich Repeat receptor-like Kinase, LRK1 [6]. In plant defense, Xa21, a LRR-RLK-type protein, was found to be the cardinal candidate for resistance to bacterial blight pathogen in rice [7]. Other defense-related candidates detected in rice include benzothiadiazole (BTH)-induced SERK 1 (OsBISERK1 [8]), Blast Resistance-Related protein (OsBRR1 [9]), and Somatic Embryogenesis Receptor-like Kinase (OsSERK [10]). In addition to these, dual function or multiple ligand-receptor recognition may be involved in interactions between disease and developmental pathways. For example, LRR-RLK ERECTA was experimentally determined to be involved in both developmental and defense systems in *Arabidopsis thaliana* [11]. Based on the few characterized plant defense-related genes, it appears that the LRR region serves as a specific recognition site for pathogen gene products, followed by a series of signal transduction cascade and activated defense mechanisms [12]. Despite its importance in plant defense pathways, the *LRR-RLK* type of candidate resistance genes and their products have however yet to be assessed in rice.

The common wild rice, *Oryza rufipogon* Griff. (Poaceae, AA genome, 2*n* = 24), is an important source of genes in rice breeding programs [13]. Although phenotypically inferior to modern cultivated rice, breeders have long recognized the intrinsic value of *O. rufipogon* genes in rice breeding [14,15]. By using diverse crosses, defense-related genes and QTLs associated with these disease resistance traits have been placed on genetic linkage maps (e.g., resistance to Tungro virus [16] and blast disease resistance [17]). Among the wild rice varieties, a Malaysian accession of *O. rufipogon* (IRGC105491) has been used for mapping and cloning of genes underlying a red-pericarp gene [18], flowering time [19], seed size [20], plant stature [21] and yield per plant [22]. The extent of genetic polymorphism and phenotypic variation is however as yet unknown for the *O. rufipogon* IRGC105491 disease resistance genes.

Previously, we characterized the wild rice yield enhancing QTL *yld1.1* region by comparative sequencing of *O. sativa* and *O. rufipogon* around the closely linked simple sequence repeat (SSR) RM5 [23], which has been repeatedly reported to be linked to yield enhancing QTL *yld1.1* from different genetic backgrounds [14]. As a healthy defense system plays an active role in rice grain development and can be expected to ultimately promote yield potential [24], it is reasonable to study potential defense-related genes located within the characterized *yld1.1* region. Hence, we set out to further investigate an *LRR-RLK* gene **(**designated as *OrufRPK1*), which is one of fourteen predicted genes located within the *yld1.1* region [23]. Besides the potentially defense-related LRR features of *OrufRPK1*, a gene cluster including eight *LRR-RLK* genes has been reported by Zha, *et al*. [6] as a candidate involved in improving rice yield performance. In this study, we report the structural organization and expression pattern of the *OrufRPK1* gene in *O. rufipogon* and describe comparative analysis of the gene among three selected cultivars of *O. sativa* (*O. sativa* ssp. *indica* cv. MR219, *O. sativa* ssp. *indica* cv. 9311 and *O. sativa* ssp. *japonica* cv. Nipponbare) and *O. rufipogon* IRGC105491. Our findings provide interesting insight into the functional roles of the *OrufRPK1* and its orthologs in wild and cultivated rice.

## 2. Results and Discussion

### 2.1. Results

**Figure 1:**
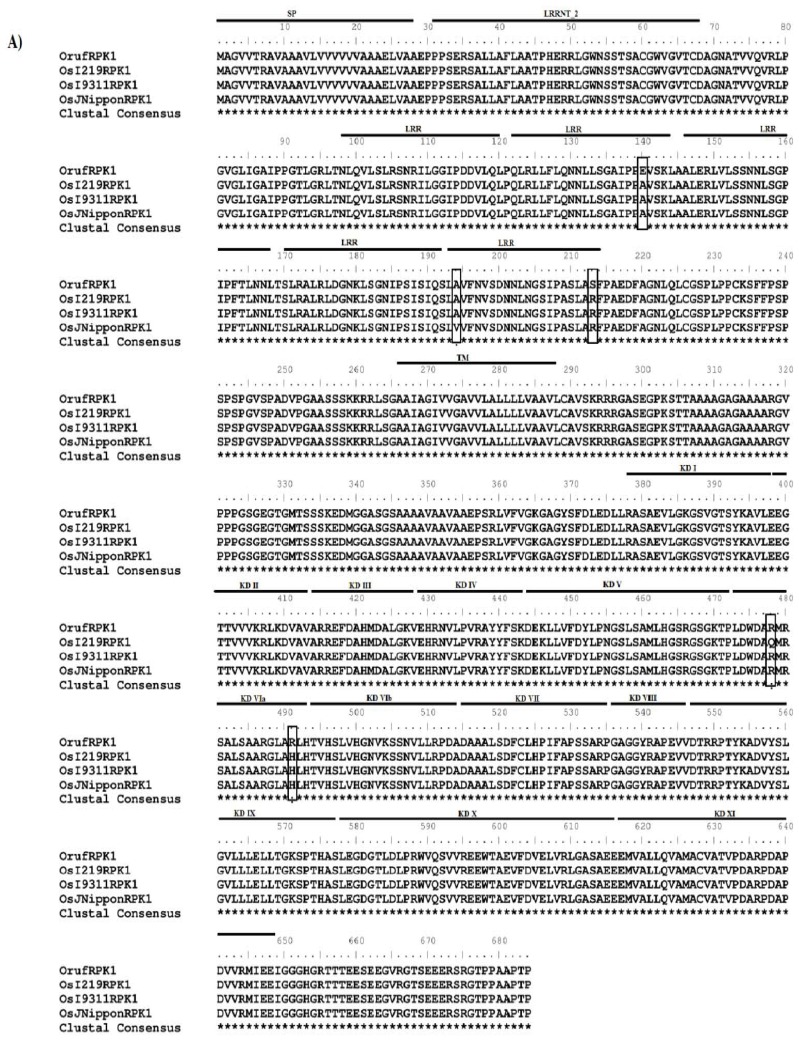

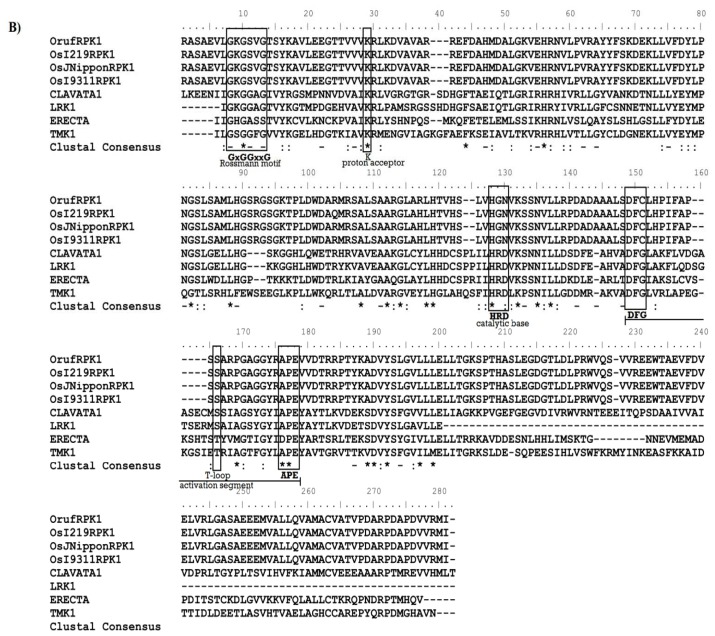
Comparison of OrufRPK1 with orthologous proteins. (**A**) Amino acid sequence alignment of OrufRPK1, OsI219RPK1, OsI9311RPK1 and OsJNipponRPK1. All five amino acid substitutions are vertically boxed. SP: signal peptide; LRR: leucine-rich repeat; TM: transmembrane domain; KD: serine/threonine kinase domain; (**B**) ClustalW amino acid alignment of kinase domains with known RLKs. OrufRPK1 (GI: 380710170; 382–643) from *Oryza rufipogon*; OsI219RPK1 (GI: 380710172; 382–643), OsI9311RPK1 (GI: 218188631; 382–643) OsJNipponRPK1 (GI: 53792194; 382–643) and LRK1 (GI: 8132685; 705–894) from *Oryza sativa*; CLAVATA1 (At1g75820; 692–968), ERECTA (At2g26330: 653–910) and TMKL1 (At3g24660; 383–569) from *Arabidopsis thaliana*.

**Figure 2:**
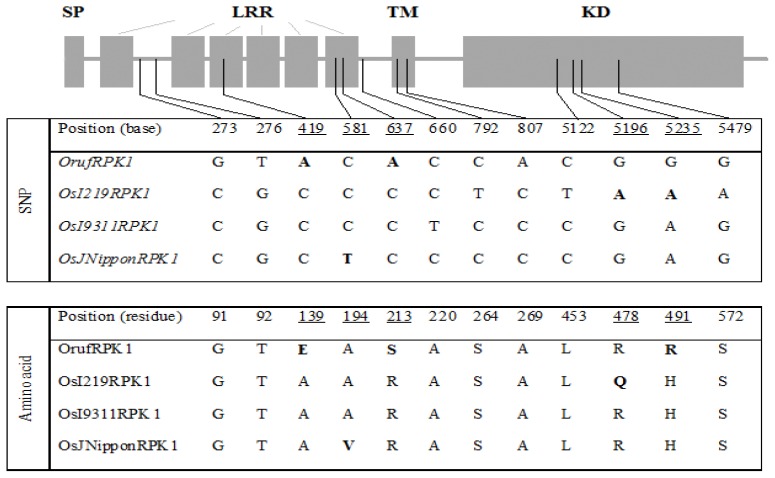
Structure of RPK1 and comparison of SNPs among four rice lines. Positions of the five substituted amino acids within the *RPK1* locus are underlined and the corresponding SNPs and amino acids are in bold text. SP, signal peptide; LRR, leucine-rich repeat; TM, transmembrane domain; KD, serine/threonine kinase domain.

Full-length cDNAs were isolated for the *RPK1* from *O. rufipogon* (accession IRGC105491, designated as *Oryza rufipogon receptor-like protein kinases 1*, *OrufRPK1*; Genbank accession number GI: 380710170) and *O. sativa* ssp. *indica* cv. MR219 (named as *Oryza sativa ssp. indica cv.* MR219 *receptor-like protein kinases 1*, *OsI219RPK1*; Genbank accession number GI: 380710172). The coding regions of the *RPK1* genes from the two rice species were identical in length (2055 bp) whilst the 5′ UTR of *OrufRPK1* and *OsI219RPK1* were 404 bp and 103 bp respectively and the 3′ UTR were 386 bp and 418 bp in length. A putative polyadenylation signal site was found in the presumptive 3′ UTR region of each cDNA. Alignment of each cDNA with the corresponding genomic sequences found that all exon-intron boundaries obey the canonical splice donor-acceptor rule (GT-AG). The length of the intron in *OrufRPK1*, *OsI219RPK1*, *OsI9311RPK1* and *OsJNipponRPK1* was found to be varied: 3752 bp, 3372 bp, 3345 bp and 3357 bp, respectively (Table 1). Analysis of the coding regions revealed the first start codon (ATG) in exon 1 and the first stop (TGA) located at exon 2. Both *OrufRPK1* and *OsI219RPK1* were found to share same genomic location of the ATG and TGA sites. Analysis of structural properties of the RPK1 proteins using SMART program [25,26] suggests that all of the four RPK1 sequences contain a predicted signal peptide, 6 extracellular LRR repeats, a transmembrane domain and a cytoplasmic serine/threonine kinase domain (Figures 1 and 2).

**Figure 3:**
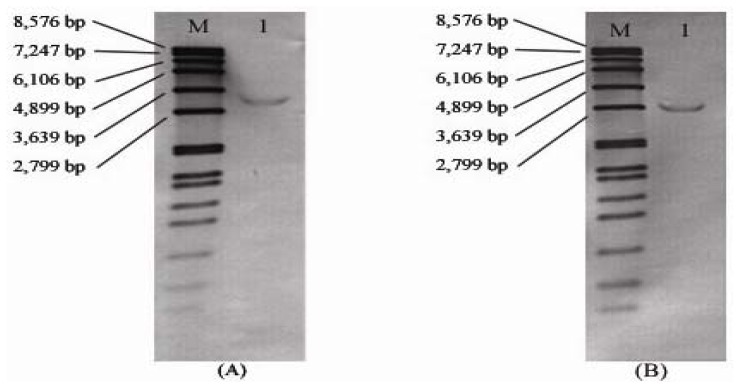
Southern analysis of (A) *O. rufipogon*; (B) *O. sativa* ssp. *indica* cv. MR219 genomic DNA probed with a 320 bp 3′ UTR fragment of *RPK1*. Lane M contains a DIG-labeled DNA Molecular Weight Marker VII (Roche, Germany). Lane 1: genomic DNA digested with *Eco*RI.

Southern-blot hybridization of rice genomic DNA using a 320 bp 3′ UTR fragment as probe indicated that only a single *RPK1* copy exists in the genome of *O. rufipogon* and *O. sativa* ssp. *indica* cv. MR219 (Figure 3). As expected from restriction site analysis of the sequences, *Eco*RI fragments of approximately 3.3 kb and 2.9 kb hybridized with the 3′ UTR probe in the wild and cultivated genomes, respectively, consistent with the different intron sizes predicted from the sequence analysis. In marked contrast, the analysis revealed only weak signals for *Bam*HI and *Hin*dIII restriction fragments, which were all larger than 8 kb in size (data not shown).

Multiple sequence alignment of the *RPK1* coding and amino acid sequence from the *O. rufipogon*, *O. sativa* ssp. *indica* cv. MR219, *O. sativa* ssp. *indica* cv. 9311 and *O. sativa* ssp. *japonica* cv. Nipponbare identified a panel of SNPs (Figures 1 and 2). From a total of 12 SNPs identified within the exonic regions, six specifically defined the *O. rufipogon* allele, while 4, 1 and 1 SNPs characterized the *indica* (MR219), *indica* (9331) and *japonica*, respectively (Figure 2). Further characterization of the degree of genotypic variation between different rice species revealed a major insertion of a 392 bp segment at genomic sequence positions 2731 to 3122 bp in the *O. rufipogon OrufRPK1*. Additionally, 22 unique InDels were detected in the wild rice counterpart (data not shown). Among the 12 SNPs detected, four were found to involve pyrimidine substitutions (C and T bases), purine substitutions (A and G bases) occurred at three sites, and the remaining SNPs involved base substitutions between the purine and pyrimidine groups (Figure 2). Five SNPs incurred non-synonymous substitutions, with three and two amino acid substitutions detected within an LRR motif and the kinase domain, respectively.

**Figure 4:**
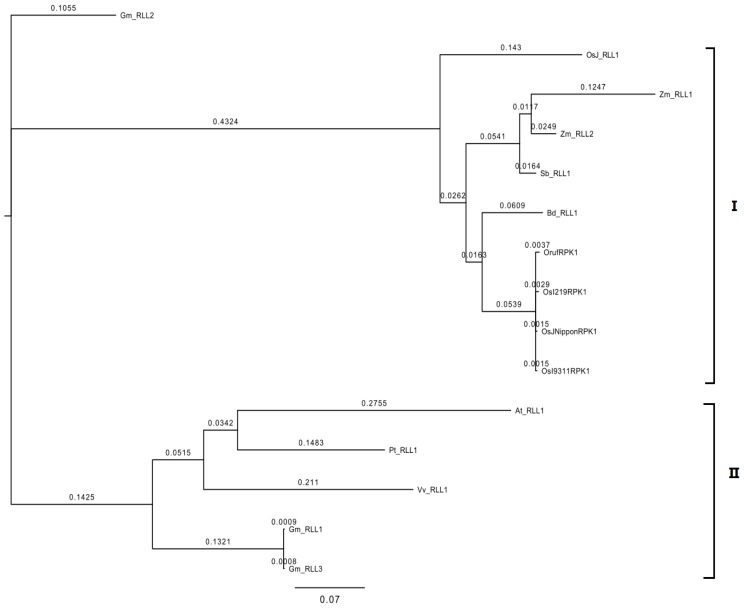
Cluster analysis of *OrufRPK1* and orthologous plant *RPK1* genes. The phylogenetic tree was constructed using maximum likelihood (MrBayes 3.2.0). Plant species abbreviations and Genbank accession numbers are as follows: At: *Arabidopsis thaliana*; Bd: *Brachpodium distachyon*; Gm: *Glycine max*; Pt: *Populus trichocarpa*; Sb: *Sorghum bicolar*; Vv: *Vitis vinifera*; Zm: *Zea mays; At_RLL1* (GI:30683225); *Bd_RLL1* (GI: 357130455); *Gm_RLL1* (GI: 357477836); *Gm_RLL2* (GI: 356516925); *Gm_RLL3* (GI: 357477836); *OsI219RPK1* (GI: 380710172); *OsI9311RPK1* (GI: 218188631); *OsJNipponRPK1* (GI: 53792194); and *OsJ_RLL1* (GI: 47777361); *OrufRPK1* (GI: 380710170); *Pt_RLL1* (GI: 280967730); *Sb_RLL1* (GI: 219885379); *Vv_RLL1* (GI: 280967730); *Zm_RLL1* (GI: 219885379) and *Zm_RLL2* (GI: 100279562).

Using the OrufRPK1 protein sequence as the search string for identification of orthologous sequences, a total of 11 orthologous *RPK1* gene sequences were identified from the Rice Kinase Database [27,28], Rice Genome Annotation Project [29,30] and Gramene Database [31,32] as shown in Table 2. Phylogenetic analysis revealed two well-supported groups (designated as group I and group II) of orthologous *RPK1* genes (Figure 3). The *OrufRPK1*, *OsI219RPK1*, *OsI9311RPK1* and *OsJNipponRPK1* grouped together with *BD_RLL1*, *SB_RLL1*, *Zm_RLL1*, *Zm_RLL2* and *OsJ_RLL1* in group II (Figure 4). The group II could be subdivided into three branches, with the *OrufRPK1* grouped along with *OsI219RPK1* and *OsJNipponRPK1* in a strongly supported (100%) cluster. In addition, the topology of the maximum likelihood method generated from these sequences is in agreement with the previously reported classification grouping of most of the orthologous *RPK1* genes to the *LRR* III families of *RLK* genes [33].

**Figure 5:**
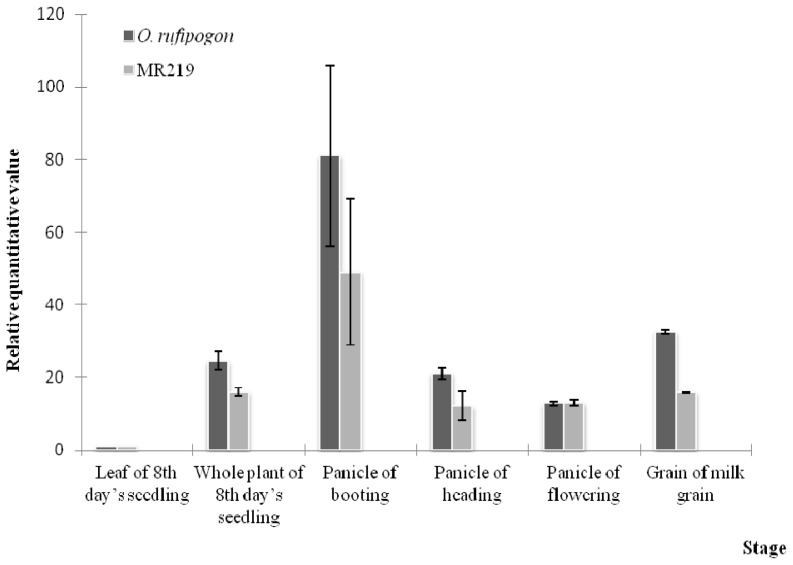
Comparative gene expression of *OrufRPK1* and *OsI219RPK1* during vegetative, reproductive and ripening phase. *O. rufipogon* and *O. sativa* ssp. *indica* cv. MR219 leaves and the whole plant were sampled at the seedling stage, the panicles at the booting, heading and flowering stages, and grains at the milk grain stage. Error bars indicate standard error of the means.

The expression patterns of *OrufRPK1* and *OsI219RPK1* were analyzed in various tissues from five different developmental stages (Figure 5). During the reproductive booting phase, *RPK1* expression in the panicle tissue of both species was significantly higher than at other developmental stages. A relatively lower expression level was observed in MR219 when compared to the wild rice (relative quantitative value 50, as compared to 81 in *O. rufipogon*, Figure 5). Overall, *OrufRPK1* transcripts had higher expression levels across all developmental stages. After the booting stage, gene expression gradually decreased until the flowering stage. The expression of *OrufRPK1* of the milk grain stage coincides with the onset of the ripening phase. Statistical analysis using two-way ANOVA with rescaled normalized expression level from the *RPK1* of *O. rufipogon* and *O. sativa* ssp. *indica* cv. MR219 collected at different rice development stages, revealed significant differences (*p* < 0.05) between species and between stages within each species.

**Figure 6:**
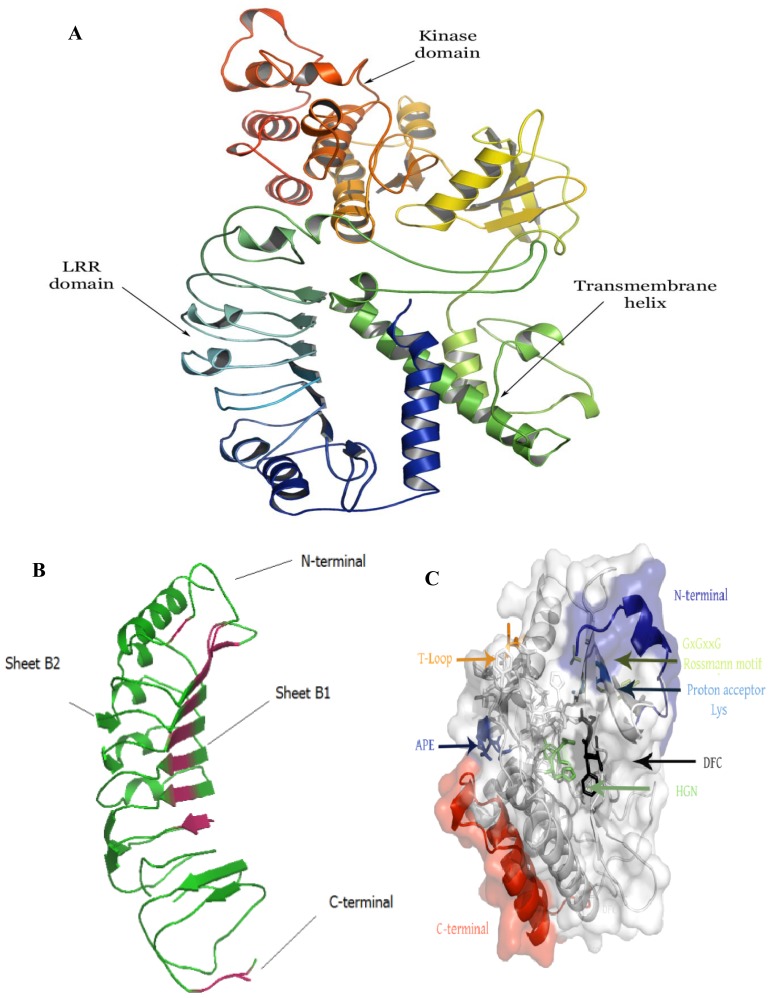
Predicted three dimensional structure of the OrufRPK1 protein. (**A**) The predicted OrufRPK1 protein structure contains an LRR domain (blue), kinase domain (red) and transmembrane helix (yellow); (**B**) The LRR domain of OrufRPK1 consists of β-sheets and helices within N and C terminals; (**C**) Several highly conserved residues highlighted within the kinase domain of the OrufRPK1 protein.

The three dimensional structure of OrufRPK1, OsI219RPK1, OsI9311RPK1 and OsJNipponRPK1 proteins were modeled using Robetta webserver (Figure 6 and Figure S1, Supplementary) [34]. The modeled OrufRPK1 protein possesses two main domains, the LRR domain and kinase domain (Figure 6). Structural assessment by Ramachandran plot predicted the structure to have 83.4% of residues in the core region and the rest of the residues within allowed regions. The predicted three dimensional structure of OrufRPK1 LRR domain contains similar unique features with the comparative modeling template, polygalacturonase-inhibiting protein, which contains four LxxLxLxxN motifs (Figure 6b). The secondary structure of the LRR domain of the OrufRPK1 protein is distributed as Helixes (α + 3/10) and β-strands (β1 and β2), together comprising of 35% of the structure with the remainder forming turns and bends, which are responsible for the curving nature of LRR proteins (Figure 6b) [35]. Figure 6c shows several highly conserved residues within the kinase domain of the OrufRPK1 protein, including a Rossmann motif, protein acceptor and activation segment.

### 2.2. Discussion

Plant LRR-RLKs are known as cell surface reporters which regulate developmental and defence related signal transduction pathways. In rice, a model which represents monocots, 380 LRR-RLK encoding sequences have been identified in the genome [36], almost twice the number found in Arabidopsis. However, only a small fraction of the rice LRR-RLK members have been functionally investigated or molecularly characterized. The LRR-RPK-encoded *OrufRPK1* gene characterized in the present study was partially mapped in previous work using an *ab initio* gene prediction method [23]. However, high-accuracy identification of promoter, transcriptional start site and terminal exons remains difficult for *ab initio* approaches. Here, we employed direct mapping of the sequenced *OrufRPK1* cDNA to its genomic sequence and performed comparative analysis with orthologs in cultivated rice genomes.

#### 2.2.1. Structural Prediction of *OrufRPK1* and *OsI219RPK1*

For RPK1 orthologs *OrufRPK1 and OsI219RPK1* we determined coding sequences of 2055 bp forming two exons contained within 5807 bp and 5427 bp genomic regions respectively (Table 1). Southern blot analysis confirmed that both *O. rufipogon* and *O. sativa* ssp. *indica* cv. MR219 contain a single copy of the *RPK1* gene and the *RPK1* 3′ UTR probe revealed a size difference between the *Eco*RI fragments, consistent with the intron size difference between the two *RPK1* alleles. The encoded RPK1 protein consists of LRR extracellular domains, a transmembrane helix and an intracellular cytoplasmic protein kinase (Figure 5). OrufRPK1 ortholog, OsJNipponRPK1, has been assigned to the LRR III subfamily of LRR-RLKs by Jung, *et al*. [37]. According to Shiu and Bleecker [38], the LRR III subfamily is known as atypical RLK because most of their sequences lack the aspartic acid in the kinase subdomain VIb.

Kinase domains are responsible for the phosphorylation of substrates by transferring the γ-phosphate of ATP to a hydroxyl moiety after activation [39]. The consensus Rossmann motif GxGxxG and an invariant lysine (Lys) were identified within the OrufRPK1 kinase subdomain I and kinase subdomain II respectively (Figure 1b and Figure 6c). As reviewed by Dissmeyer and Schnittger [40], a Rossmann motif located within a phosphate binding loop (P-loop) helps to anchor ATP, while the invariant Lys (K) assists in the enzymatic process of phosphate transfer. However, the His-Gly-Asn (HGN) and Asp-Phe-Cys (DFC) motifs were identified within RPK1 kinase subdomain VIb and kinase subdomain VII respectively instead of conserved residues His-Arg-Asp (HRD) and Asp-Phe-Gly (DFG) motifs (Figures 1b and Figure 6c) as would be expected for a kinase catalytic site and ATP binding respectively. Structural alignment of the OrufRPK1 kinase domain DFC and DFG motifs (Supplementary, Figure S2a), show the DFC motif (magenta) to contain three hydrogen bonds at the activation loop, whilst, the DFG motif (green) contains two conserved hydrogen bonds to form the 3-turn between DFG phenylalanine, DFG glycine and the other two residues, a characteristic feature of activation loop in active state of the kinases [41]. The missing conserved 3-turn in the OrufRPK1 kinase domain is due to a cysteine in place of a glycine that would be expected in a conserved DFG motif. Structural alignment between the HGN motif of kinase domain VIb OrufRPK1 with that of the conserved domain form RD kinases, showed that OrufRPK1 lacks the conserved amino acids RD. This may affect the formation of hydrophobic interactions with the conserved DFG motif (Supplementary, Figure S2b). These findings raise the possibility that the OrufRPK1 is a catalytically inactive atypical kinase, thus cation binding and orientation of the ATP γ phosphate for phosphate transfer may be retarded [42]. According to Kornev, *et al*. [41], the glycine of the DFG motif is an important invariant amino acid in kinase domains and it plays a role in the bipositional switch that reorients the aspartate of DFG into an active or inactive conformation. Modification of the DFG motif to a DFC motif as in the OrufRPK1 kinase domain may result in a lack of this bipositional switch. Based on these predictions, the kinase domains of OrufRPK1 and OsI219RPK1 proteins are atypical kinase domains which would be involved in phosphorylation-independent mechanisms in signal transduction.

#### 2.2.2. Inter- and Intra-Specific *OrufRPK1* Sequence Comparison

Based on the predicted 3D structure of OrufRPK1, the positions of the identified non-synonymous SNPs are found to correspond with minor or no change in the predicted protein structures among the four rice lines compared (Supplementary, Figure S1). However, one of the two SNPs associated with the non-synonymous amino acid mutations in the kinase conserved domain, position 478, results in replacement of a positively charged arginine (R) residue (OsJNipponRPK1 and OrufRPK1) with a neutral glutamine (Q) residue in OsI219RPK1 (Figure 2). This substitution confers a gain of charge to the amino acid residue in OsI219RPK1 thus may alter the catalytic functions and contribute to functional diversity among the orthologs of RPK1.

#### 2.2.3. Possible Role of OrufRPK1 and OsI219RPK1: A Defense Story

The high similarity (99% identity over 684 amino acids) between the rice RPK1 sequences together with the phylogenetic grouping of the OrufRPK1 amino acid sequence with OsI219RPK1, OsJNipponRPK1 and OsI9311RPK1 (Figure 4), suggests that these rice orthologs may have the same function. Real time quantitative RT-PCR (qRT-PCR) expression analysis revealed similar expression patterns for both *OrufRPK1* and *OsI219RPK1* with very low expression at the seedling stage, especially in leaf, with 3–4 fold up-regulation in panicles at the booting stage and a gradual decrease of expression over the following phases, as would be expected if they play similar roles in both species. The gene expression profiles of *OrufRPK1* and *OsI219RPK1* were also similar to those reported for rice disease resistance gene *Xa3* [43] and it is notable that both rice disease resistance genes *Xa3* and *Xa21*, against bacterial pathogen *Xanthomonas oryzae* pv. *oryzae*, show increased expression in adult compared to juvenile plants [43,44]. Based on a rice interactome network survey, Gu, *et al*. [45] proposed that OsJNipponRPK1 interacts with the pathogen-related calmodulin like proteins such as LOC_Os03920370.1 (Cytoplasm), LOC_Os01g16240 (Nucleus), LOC_Os05g41210.1 (OsCAM2) and LOC_Os07G48780 (Oscam 1–2). In addition, previous functional analysis of rice bacterial blight resistance gene *Xa26* [46], showed that the LRR domains subjected to positive selection, strongly correlated with those that contribute to pathogen recognition specificities. Interestingly, window sliding analysis suggested that the position between 200 and 230 amino acids of OrufRPK1, corresponding to the LRR domain (residues 192–214) has been subjected to positive selection (Supplementary, Figure S3). This is due to a non-synonymous amino acid substitution of Arg^213^ to Ser^213^ in OrufRPK1. The predicted 3D structure of OrufRPK1 in the present study revealed four out of six predicted LRR domains to have a concave structure, consisting of four LxxLxLxxN motifs (amino acids 99–110 LQVLSLRSNRIL; amino acids 123–133 LRLLFLQNNLL; amino acids 147–157 LERLVLSSNNL and amino acids 171–181 LRALRLDGNKL) at parallel arranged β-sheets (β1 and β2) linked by adjacent turns (Figure 6b). In general, β-sheets located in the LRR domain are the main site of protein interaction and ligand binding [47,48]. Although one of the LRR domains (residues 192–214) of OrufRPK1 located within the positive selection region does not include an LxxLxLxxN motif, it is quite possible that this LRR domain will interact with the nearby LRR domain (residues 170–191) containing the LxxLxLxxN motif. It has been reported that there is positive selection on the LxxLxLxxN β-strand motif in LRR domains in various proteins in arabidopsis and rice, suggesting that the β-strand motif is involved in diversifying selection [49,50]. Therefore, these results are consistent with the LRR domain of OrufRPK1 contributing to ligand binding and/or receptor specificity in disease resistance pathway, under positive selection and stress environment.

## 3. Experimental Section

### 3.1. Plant Materials

Seeds of *O. rufipogon* accession IRGC105491 and *O. sativa* ssp. *indica* cv. MR219 were obtained from the Malaysian Agricultural Research and Development Institute (MARDI) Rice Genebank, Seberang Perai, Malaysia. All plants were grown under 12 h light/12 h dark at 28 °C and 75% relative humidity in a glasshouse. Leaves and whole plants (for 8 day old seedlings), panicle (at booting, heading and flowering) and grain (milk grain stage) were collected for RNA extraction.

### 3.2. RNA Extraction and cDNA Synthesis

Total RNA was isolated during the vegetative (leaf and whole plant), reproduction (booting, heading and flowering panicle) and ripening (milk grain) phases [51]. Concentration of total RNA was measured by spectrophotometer (Eppendorf, Germany). The integrity of total RNA extracted was confirmed by 1% (*w*/*v*) agarose gel electrophoresis. Only samples with 260/280 nm values between 1.9 and 2.1 and 260/230 nm values greater than 2.0 were used for cDNA synthesis by High-Capacity cDNA Reverse Transcription Kits (Applied Biosystems, USA) according to the manufacturer’s instructions.

### 3.3. Cloning of the Full-Length *OrufRPK1*

FirstChoice^®^ RLM-RACE Kit (Ambion, USA) was used to amplify the full length cDNA of the *OrufRPK1* sequence. The rapid amplification of 5′ cDNA ends reactions were performed with 10 μg total RNA. The RNA processing and 5′ adaptor ligation for cDNA synthesis was performed according to the manufacturer’s instructions. The outer 5′ RLM-RACE PCR was performed using 5′ RACE gene-specific outer primer: 5′-AACTGCAGCGGATACGACCACAC-3′ and 5′ RACE outer primer; while the inner 5′ RLM-RACE PCR was performed with a 5′ RACE gene-specific inner primer: 5′-TGCAGCGGATACGACCACACT-3′ and a 5′ RACE inner Primer. For the rapid amplification of 3′ cDNA ends, 1 μg total RNA was used. The 3′ adaptor ligation for cDNA synthesis was performed according to the manufacturer’s instructions. The outer 3′ RLM-RACE PCR was performed with a 3′ RACE gene-specific outer primer: 5′-AGGTTCTTTGGCTGCCTGTATG-3′ and a 3′ RACE outer primer; while the inner 3′ RLM-RACE PCR was performed with a 3′ RACE gene-specific inner primer: 5′-TCATCGTCTGCCCCCTTTGTG-3′ and a 3′ RACE inner primer. The 5′ and 3′ RLM-RACE PCRs were performed using AccuPrime™ GC-Rich DNA Polymerase (Invitrogen, California). PCR products were analyzed by 1% agarose gel electrophoresis, and then cloned using a CloneJET™ PCR Cloning Kit (Fermentas, Lithuania).

### 3.4. Sequence Analysis

Gene sequences were analyzed using GenScan [52,53]. The predicted peptide sequences were analyzed using Simple Modular Architecture Research Tool [25,26]. The kinase domains of OrufRPK1, OsI219RPK1, OsI9311RPK1 and OsJNipponRPK1 were identified by SMART analysis. The signal peptide was predicted using SignalP 4.0 Server [54,55]. The transmembrane helix was predicted using Transmembrane Protein Topology with a Hidden Markov Model (TMHMM) Server 2.0 [56,57]. The LRR domain and protein kinase domain were predicted using protein domain families (Pfam) database [58]. The sequences of protein kinases, LRK1 (GI: 8132685), CLAVATA1 (At1g75820), ERECTA (At2g26330) and TMKL1 (At3g24660), were aligned with OrufRPK1 (GI: 380710170), OsI219RPK1 (GI: 380710172), OsI9311RPK1 (GI: 218188631) and OsJNipponRPK1 (GI: 53792194) using ClustalW [59,60]. The characterization of the rice kinase was based on the presence or absence of conserved amino acids, arginine and aspartic acid (D), in kinase subdomain VIb and aspartic acid in kinase subdomain VII [61].

### 3.5. Southern Hybridization

Southern hybridization analysis was used to confirm presence of single copy of the *RPK1* gene in both wild and cultivated rice genomes. Total genomic DNA of genomic DNA from *O. rufipogon* and *O. sativa* cultivar MR219 were digested separately using *Eco*RI, *Bam*HI and *Hin*dIII and fractionated in agarose gel. Digested DNA was Southern blotted to a nylon membrane, and hybridized with a 320 bp probe derived from the *RPK1* 3′ UTR region. All blotting procedures and immunological detection were carried out according to the DIG DNA labeling and detection Kit application manual (Roche, USA).

### 3.6. Real Time qRT-PCR

Primer Express 2.0 software (Applied Biosystems, USA) was used to design all primers and facilitate real time qRT-PCR analysis. Applied Biosystems 7300 Real Time PCR System was used to perform relative quantitative (Comparative CT Method) real time qRT-PCR experiments. A total of four replicates of each stage of each line were used for each gene specific primer set: *OrufRPK1*-F and *OsI219RPK1-*F: 5′-AGGTTCTTTGGCTGCCTGTATG-3′, *OrufRPK1*-R and *OsI219RPK1-*R: 5′-AGG TTCTTTGGCTGCCTGTATG-3′, and housekeeping gene primer sets [62]: *eEF-1α-*F: 5′-TTTCACTC TTGGTGTGAAGCAGAT-3′, *eEF-1α-*R: 5′-GACTTCCTTCACGATTTCATCGTAA-3′, *UBQ5*-F: 5′-ACCACTTCGACCGCCACTACT-3′, *UBQ5*-R: 5′-ACGCCTAAGCCTGCTGGTT-3′. Each 50 ng cDNA sample, was mixed with 1× SYBR Green PCR Master Mix (Applied Biosystems, USA), and 200 nM of each forward and reverse primer then added to PCR grade dH_2_O to 25 μL in an 8 well optical reaction strip (Applied Biosystems, USA). Three replicates of a negative control with dH_2_O as template were also included for each primer pair. The real time qRT-PCRs were performed under the following conditions: 1 cycle of 10 min at 95 °C, followed by 40 cycles of 15 s at 95 °C and 1 min at 60 °C. geNORM v3.4 software (Primer-Design, UK) was used to analyze relative expression of each transcript in order to obtain more accurate normalization [63]. Data were analyzed by two-way repeated measures analysis of variance (ANOVA) with *p* < 0.05 considered significant using Prism 5 (GraphPad Software, USA). Rescaled normalized expression target and standard error of rescaled normalized expression target generated from geNORM v3.4 software (Primer-Design, UK) were used for statistical analyses.

### 3.7. Phylogenetic Analysis

OrufRPK1 amino acid sequence was used for homology searches against the Rice Kinase Database [27,28,37], Rice Genome Annotation Project database [29,30] and Gramene Database [31,32]. A total of 15 orthologous *OrufRPK1* gene sequences were selected for phylogenetic analysis (Table 2). A maximum likelihood phylogenetic tree was constructed using MrBayes 3.2.0 program [64].

### 3.8. Homology Modeling of OrufRPK1 Protein

Homology modeling of OrufRPK1, OsI219RPK1, OsI9311RPK1 and OsJNipponRPK1 used a similarity based approach for modeling three dimensional proteins with Modeler Version 9v8 [65,66]. The LRR domain of polygalacturonase-inhibiting protein (1ogqA) [67] and kinase domain of interleukin-1 receptor-associated kinase 4 (2nruB) [68] templates were used for comparative modeling of the four predicted protein structures. These domains were defined using ROBETTA web server [34] which provides both *ab initio* and comparative models of protein domains [69]. Ginzu algorithm searches for homologs in various protein structures through PSI-BLAST searches were used to define domain boundaries for the given input sequence [70].

## 4. Conclusions

Together with the limited *RPK1* coding and predicted protein structure polymorphism detected among the four rice lines studied, we have shown that OrufRPK1 is phylogenetically clustered with OsI219RPK1, OsJNipponRPK1 and OsI9311RPK1, suggesting that these rice orthologs may share functional and structural similarity. This is also supported by the real time qRT-PCR expression analysis and the protein domain subfamily classification. Our findings imply that the OrufRPK1 and its counterparts from cultivated rice species are likely to be involved in plant defense signaling mechanism, supported by the fact that kinase domain architecture of these rice orthologs is typical of the *LRR-RLK* class of rice disease resistance genes. While such comparative analysis and gene expression studies would be highly informative to facilitate better understanding of the studied rice *RPK1s*, proof of their functionalities will still require appropriate mutagenesis and functional validation.

## Supplementary Information



## Figures and Tables

**Table 1:** *RPK1* genes identified in the wild and cultivated rice.

Gene name	Species name	Genbank accession no.	DNA length (bp)	cDNA length (bp)	Protein length (aa)	No. of exons	No. of introns [length (bp)]
*OrufRPK1*	*O. rufipogon*	GI: 380710170	5807	2052	684	2	1 (3752)
*OsI219RPK1*	*O. sativa* ssp*. indica* cv. MR219	GI: 380710172	5427	2052	684	2	1 (3372)
*OsI9311RPK1*	*O. sativa* ssp. *indica* cv. 9311	GI: 218188631	5400	2052	684	2	1 (3345)
*OsJNipponRPK1*	*O. sativa* ssp. *japonica* cv. Nipponbare	GI: 53792194	5411	2052	684	2	1 (3357)

**Table 2:** Summary of gene features for orthologs *OrufRPK1* and *OsI219RPK1*.

Gene name	Species	Gene locus	Genbank accession no.	Protein length (aa)
*At_RLL1*	*Arabidopsis thaliana*	AT2G26730.1	GI: 30683225	658
*Bd_RLL1*	*Brachpodium distachyon*	BRADI2G43110.1	GI: 357130455	675
*Gm_RLL1*	*Glycine max*	MTR_4g113100	GI: 357477836	655
*Gm_RLL2*	*Glycine max*	Glyma06g23590.1	GI: 356516925	653
*Gm_RLL3*	*Glycine max*	Glyma05g08140.1	GI: 356513915	625
*OsJ_RLL1*	*O. sativa* ssp. *japonica* cv. Nippobare	LOC_Os05g51070.1	GI: 47777361	710
*Pt_RLL1*	*Populus trichocarpa*	POPTR_0018s06400.1	GI: 280967730	621
*Sb_RLL1*	*Sorghum bicolar*	Sb03g027400.1	GI: 219885379	690
*Vv_RLL1*	*Vitis vinifera*	GSVIVT01015460001	GI: 280967730	653
*Zm_RLL1*	*Zea mays*	GRMZM2G050548_T01	GI: 219885379	693
*Zm_RLL2*	*Zea mays*	LOC100279562	GI: 100279562	685
