# Supplementary material for: Molecular Characterization and Comparative Sequence Analysis of Defense-Related Gene, Oryza rufipogon Receptor-Like Protein Kinase 1
Source: Int J Mol Sci. 2012 Jul 24;13(7):9343–62. doi: 10.3390/ijms13079343 (PMC3430300; doi:10.3390/ijms13079343)

Supplementary Information

Figure S1. Protein structure predictions of OrufRPK1, OsI219RPK1, OsI9311RPK1 and OsJNipponRPK1. The 3D protein structure predictions of (**A**) OrufRPK1; (**B**) OsI219RPK1; (**C**) OsI9311RPK1 and (**D**) OsJNipponRPK1. LRR domain (red), kinase domain (green) and transmembrane helix (yellow).

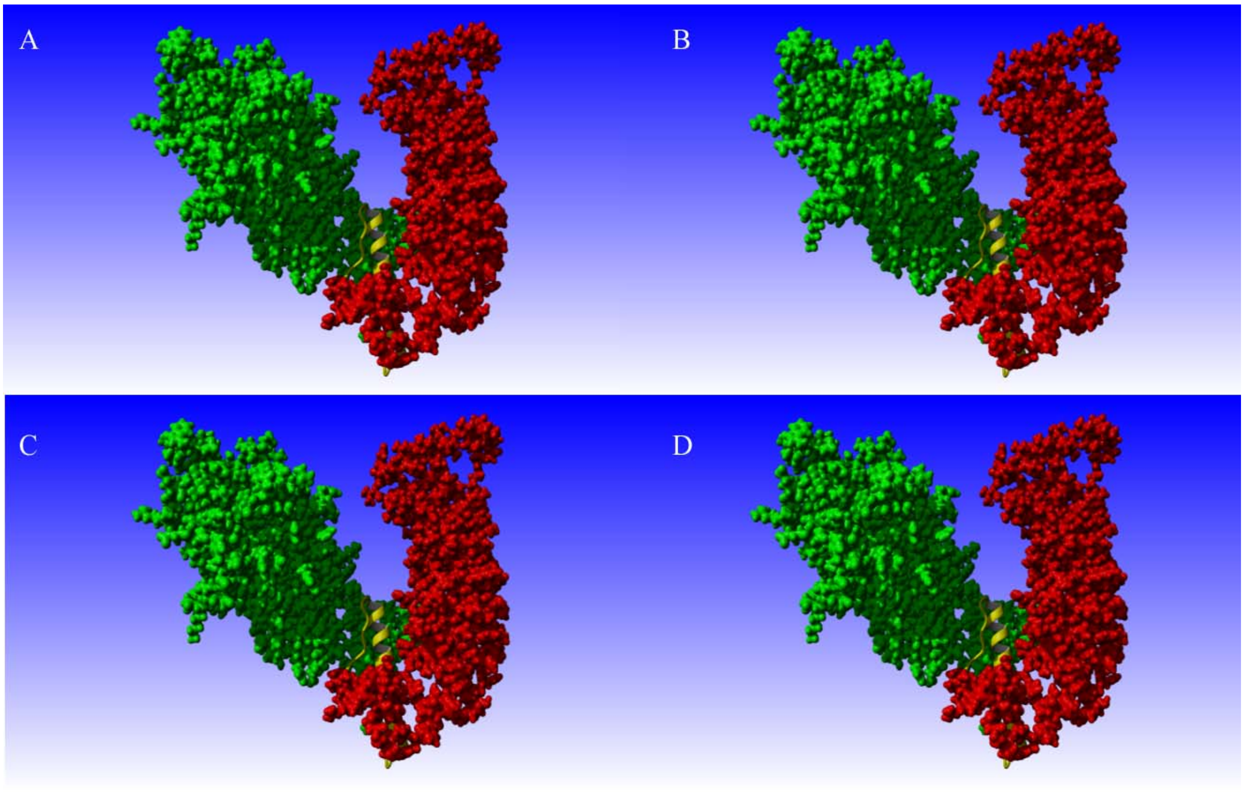

Figure S2. Structural superimposition of kinase domain of OrufRPK1 protein. Superimposition of (**A**) DFC-DFG motifs and (**B**) HRD-HGN motifs in kinase domain VII and kinase domain VIb respectively were analyzed. The DFG motif was selected from 2CPK.pdb; the HRD motif was selected from IHCL.pdb.

A

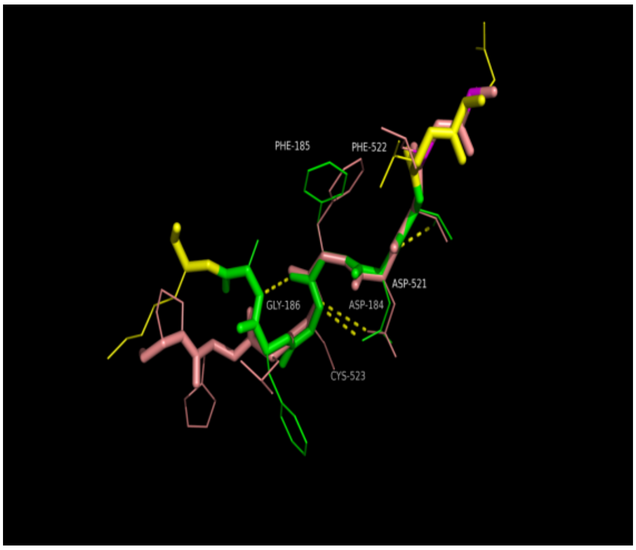

B

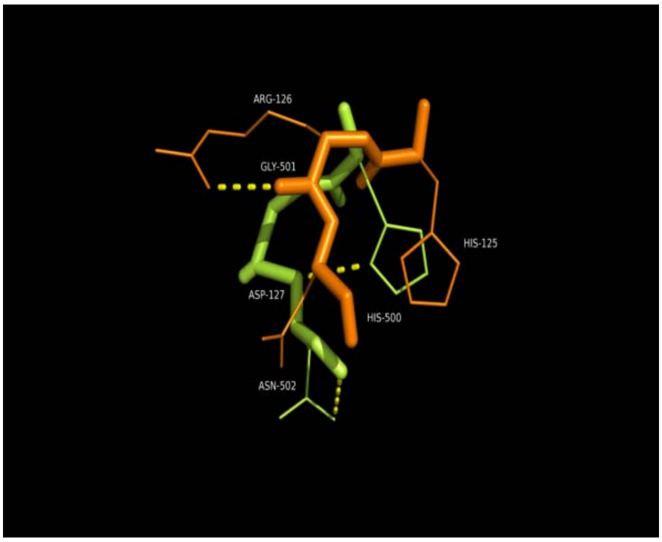

Figure S3. JCoDA output using sliding window analysis of pairwise dN/dS between *OrufRPK1*, *OsI219RPK1*, *OsI9311RPK1* and *OsNipponRPK1*. Adaptive selection dominates between amino acid 200 to 230 amino acid residues (OsNipponRPK1 versus OrufRPK1) which are overlapped with a LRR domain (amino acid 192-214). OsI219 = OsI219RPK1; OsJNippo = OsNipponRPK1; OsI9311 = OsI9311RPK1 and Oruf = OrufRPK1. All pairwise comparisons were performed using a window 200 and jump of 25.

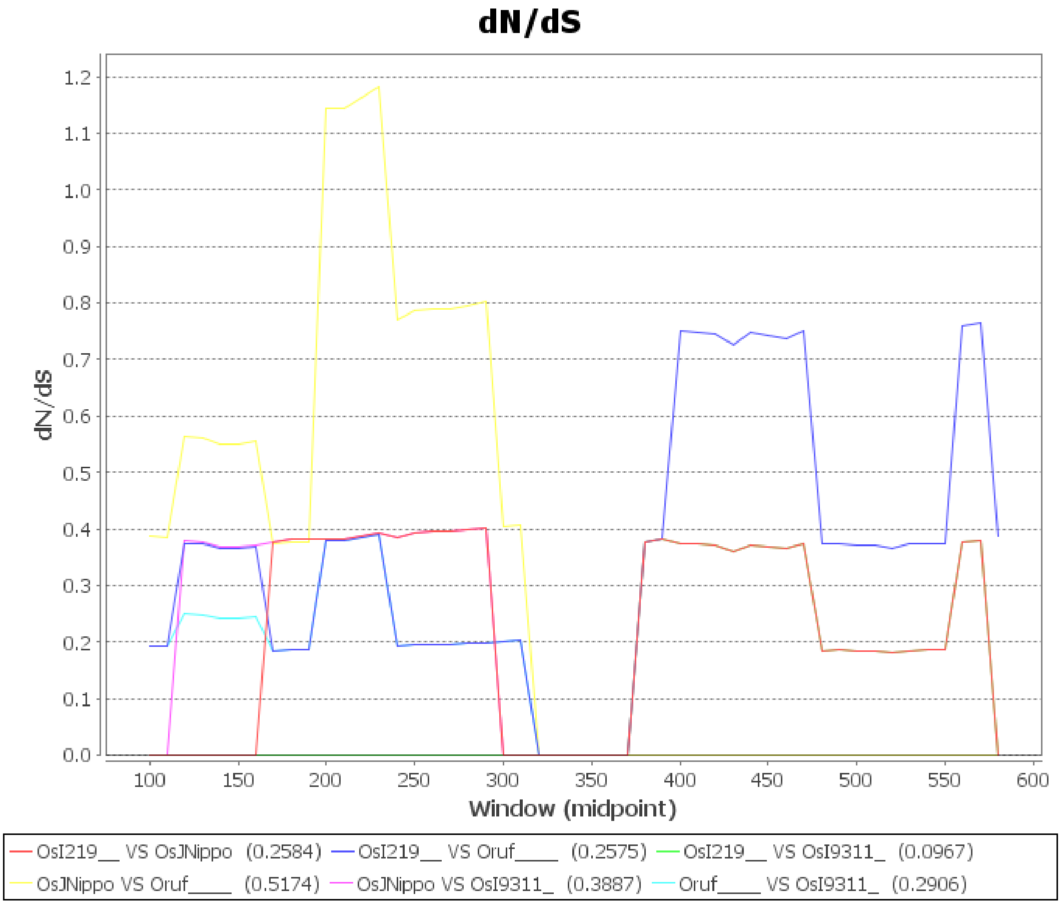

Supplement: Supplementary file 1 [file ijms-13-09343-s001.pdf]
